# Synthesis and Characterization of Novel Non-Isocyanate Polyurethanes Derived from Adipic Acid: A Comprehensive Study

**DOI:** 10.3390/polym17060728

**Published:** 2025-03-10

**Authors:** Maria Angeliki G. Ntrivala, Evangelia D. Balla, Panagiotis A. Klonos, Apostolos Kyritsis, Dimitrios N. Bikiaris

**Affiliations:** 1 Laboratory of Polymer Chemistry and Technology, Department of Chemistry, Aristotle University of Thessaloniki, GR-541 24 Thessaloniki, Greece; ntrivalamariaangeliki@gmail.com (M.A.G.N.); euagelia226@gmail.com (E.D.B.); pklonos@chem.auth.gr (P.A.K.); 2 Department of Physics, National Technical University of Athens, Zografou Campus, GR-157 80 Athens, Greece; akyrits@central.ntua.gr

**Keywords:** sustainability, non-isocyanate polyurethanes, NIPUs

## Abstract

The increasing quest for greener and more sustainable polymeric materials has gained interest in the past few decades. Non-isocyanate polyurethanes (NIPUs) have attracted attention considering that they are produced through less toxic methods compared to the conventional polyurethanes (PUs) obtained from petroleum resources and toxic isocyanates. In this context, adipic acid, glycerol carbonate, 1,2-ethylenediamine, and 1,6-hexamethylenediamine, were used to synthesize NIPU_ethyl and NIPU_hexa, respectively. The obtained NIPUs were characterized using nuclear magnetic resonance spectroscopy (H-NMR spectra) and Fourier-transform infrared spectroscopy (FTIR) analysis, which verified the structures of the intermediate and final products. Calorimetric and dielectric studies provided direct and indirect support for the facilitated thermal stability of NIPU_ethyl and NIPU_hexa. Compared to the intermediate product, the NIPUs exhibit elevated glass transition temperatures, suggesting the formation of more rigid structures. The NIPUs were also tested in terms of swelling properties, and the results indicated that NIPU_hexa absorbs and withholds increased amounts of water for longer time periods compared to NIPU_ethyl, and their hydrolysis and enzymatic hydrolysis confirmed that NIPU_hexa is more stable in aqueous environments than NIPU_ethyl. Therefore, the successful production of adipic-acid-based NIPUs through a novel perspective of the polyaddition path is reported and complemented by the characterization of the obtained materials with several techniques.

## 1. Introduction

The fact that the majority of polymers usually derives from petrochemicals seems to be contributing to two major problems that humanity is currently facing: the shortage of fossil resources and the environmental pollution, which is linked with the global warming phenomenon [1,2,3]. Simultaneously, modern industry’s overexploitation, oil shortages, and rising prices are important factors in global economic growth, while the large-scale use of fossil fuels also leads to pollution and the ecological degradation of the human living environment [2]. Therefore, researchers turn their interest toward the replacement of fossil resources with natural and sustainable ones [1,2].

Biomass feedstocks are a promising resource mainly because of their sustainability [1] and are commonly derived from trees, plants, or, alternatively, they can be biomolecular functional materials, lipid-based materials, and protein materials [2]. “Biobased polymers”, which are produced from biomass, ref. [1], are reported to have adequate biocompatibility and biodegradability, while they are additionally considered as green and environmentally friendly [2].

Polyurethanes (PUs) are a versatile class of synthetic polymeric materials, ranked as the sixth most common type of plastic worldwide [2,4]. PUs are characterized by the repetitive presence of the urethane linkage -NH-C(=O)-O- and can be considered as esters or amide esters of carbonic acid [5]. In 2018, PUs accounted for 7.9% of the total plastic end-user market (359 million tons) [4]. Furthermore, in 2020, the PU market exceeded USD 70.67 billion and is expected to reach USD 94.59 billion by 2029 [6]. Currently, polyurethanes are obtained mostly through the reaction between a polyol, which consists of multiple hydroxyl groups, along with another component containing two or more moieties of isocyanate [7].

Among their benefits, polyurethanes are versatile, with unique chemistry, excellent mechanical and optical properties, and good solvent resistance [5]. The thermodynamic incompatibility between their soft and hard segments results in the micro-phase separation structure of polyurethanes, which eventually leads to important properties, such as foamability, high elasticity, wear resistance, good low-temperature resistance, electrical insulation [2], durability, toughness, and abrasive resistance. It is also reported that these materials exhibit biocompatibility and biostability [8,9].

Polyurethanes have been extensively used in a vast range of applications, such as in packaging, construction, fabrics, coatings, foams, in the automotive industry, and even in biomedical applications [8]. More specifically, plenty of work has been conducted in the biomedical field, and the results have shown that biobased PUs can be used as wound-dressing materials in the form of foams, fibrous mats and/or films, as scaffolds for bone tissue regeneration (hard tissue), or even cartilage tissue repair (soft tissue). PUs have also been successfully tested for muscular, cardiac system, and vascular applications. Owing to their tunable compositions, they have been widely described as proper materials for the preparation of drug delivery devices and tissue adhesives [10]. A representative example is the work of Yu, Chao, et al., in which redox-sensitive micelles were obtained by the self-assembly of polyurethane and showed the controlled release of loaded doxorubicin, an anticancer drug [11].

However, despite their numerous applications, they still present certain serious disadvantages and dangers. The environmental impact of chemical substances that are used or produced during polyurethane manufacturing are evaluated through the three main stages of the polymer’s life cycle: during the synthesis of the monomers, which requires the use of toxic phosgene; during polymerization, which involves the use of CMR (carcinogenic, mutagenic, and reprotoxic)-classified isocyanates, like methylene diphenyl diisocyanate (MDI) and toluene diisocyanate (TDI); and at the end of the life of the materials, when most of the time, the two main options are either the combustion or the disposal of the polymer. During combustion, isocyanates are released and decompose into hydrogen cyanide, while in the case of disposal in a landfill, polyurethanes undergo hydrolysis and eventually yield toxic amines [6,12,13]. It can be clear that polyurethanes exhibit hazards during their whole life cycle, mainly because of the presence of harmful precursors, like isocyanates and phosgene. Isocyanates, especially, are reported to be progressively banned in Europe, with REACH regulations [12]. Consequently, there is an increasing urge to start synthesizing polyurethanes without the use of isocyanate molecules.

Non-isocyanate polyurethanes (NIPUs) can be produced via four synthetic routes: the polycondensation route, the rearrangement route, the ring-opening polymerization route, and the polyaddition route. The first three routes present similar disadvantages, such as the fact that they utilize toxic reagents, like phosgene derivatives, acyl azides, or carboxamides. Furthermore, these synthetic paths generate undesired byproducts (for example, alcohols or hydrochloric acid, HCl), and they also require extreme temperature conditions. Hence, the favored route would be polyaddition, which includes the reaction of diamines with (most of the time, five-membered) cyclic carbonates [6].

NIPUs are considered to be reliable and exceptionally promising materials for an extensive number of applications, including biomedical applications. NIPUs are reported to be suitable as wound-dressing materials with antibacterial activity [14], as resins for the 3D printing of customized biocompatible orthopedic surgical guides [15], as elastomers for biomedical implants in the cardiovascular field [16], and as composite materials, along with chitosan and cellulose nanofibers, in the form of porous, 3D-printable hydrogels for antibacterial wound-healing applications [17]. They have also been studied as possible biomaterials for the manufacturing of improved prosthetic heart valves, and the results were described as promising [18]. Furthermore, NIPUs seem to have potential utility in drug delivery [19]. For example, Pramanik, Sreedharan et al. have developed non-isocyanate-based polyurethane nanocapsules that target mitochondria for the enzyme-triggered release of rhodamine and doxorubicin [20].

In the present work, we describe the synthesis of two non-isocyanate polyurethanes, NIPU_ethyl and NIPU_hexa, which are synthesized using 1,2-ethylenediamine and 1,6-hexamethylenediamine, respectively. Their synthesis was completed in two steps. The first step consists of the reaction between adipic acid and glycerol carbonate to form an ester molecule (intermediate product). The intermediate product is then used for the second step of the synthetic route, where, after purification, it reacts with a diamine, in our case, 1,2-ethylenediamine or 1,6-hexamethylenediamine. According to the existing literature, adipic acid can be obtained through the chemo-catalytic pathway in which biomass-derived glucose is aerobically oxidized to glucaric acid, a molecule that eventually undergoes hydrodeoxygenation to yield adipic acid [21]. Glycerol carbonate can also be obtained from biomass through various pathways starting from glycerol acquired by the biodiesel industry. The main promising route involves the fixation of CO_2_ [22]. The employed diamines are also reagents with the ability to be attained from the products of biomass. For instance, starting from adipic acid and through the production of adiponitrile as an intermediate, 1,6-hexamethylenediamine is produced via catalytic hydrogenation [23]. Consequently, all the materials used in the present study can be obtained from the renewable resource of biomass, and, therefore, the products of our present work could be biobased. The authors would like to clarify that although the present study focuses on NIPU synthesis from biobased materials, the reagents used were of analytical grade to ensure accurate and reproducible results. The aforementioned approach can establish a basis of data in order to apply the findings to biobased materials. For the investigation of the structure and properties of the obtained NIPUs, a variety of techniques was employed, including nuclear magnetic resonance spectroscopy (NMR), Fourier-transform infrared spectroscopy (FTIR), differential scanning calorimetry (DSC), thermogravimetric analysis (TGA), and broadband dielectric spectroscopy (BDS). Additionally, their capacities in swelling, solubilities at various pH levels, and dehydration abilities were also studied. Although many research articles have been published on the synthesis of polyhydroxy urethanes (PHUs), including those from diacids, to the best of our knowledge, there are not any scientific studies on adipic-acid-based NIPUs synthesized through the polyaddition route using glycerol carbonate and either 1,2-ethylenediamine or 1,6-hexamethylenediamine.

## 2. Materials and Methods

### 2.1. Materials

Adipic acid (A.A., 99%, Alfa Aesar, Haverhill, MA, USA), 4-hydroxymethyl-1,3-dioxolan-2-one (glycerol carbonate, G.C., 90%, Fluorochem, 14 Graphite Way, Hadfield, Glossop, UK), 4-((N,N‣-dimethylamino)pyridine) (DMAP, 99%, Alfa Aesar), N,N‣-dicyclohexylcarbodiimide (DCC, 99%, Alfa Aesar), 1,2-ethylenediamine (98%, Merck, Rahway, NJ, USA), 1,6-hexamethylenediamine (98%, Merck), sodium sulfate anhydrous (Na_2_SO_4_ anh., Lachner, Tovární, Neratovice, Polland), dichloromethane (DCM, ≥99.8%, analytical reagent grade, Fisher, Hampton, NH, USA), and hydrochloric acid (HCl, ChemLab, Zedelgem, Belgium) were used.

### 2.2. Synthesis

#### Synthesis of Adipic-Acid-Based NIPUs

The synthesis reaction took place following the polyaddition route according to the work published by Jaratrotkamjorn, Ruedee et al. [13]. For this purpose, 2.5 g of previously dried A.A. (0.017 mol) (60 °C, vacuum) was added to a round flask along with 1.5 eq. (0.025 mol) of DMAP, 3 eq. (0.051 mol) of DCC, and 3 eq. (0.051 mol) of G.C. The G.C. was added dropwise with a syringe that punctured the septum, which kept the flask closed. The solvent (DCM) was then added dropwise inside the flask until reaching a total volume of 30 mL. The procedure was repeated three times. The reaction took place under constant stirring and in a N_2_ atmosphere for 24 h. Because of the DMAP-DCC-DCM employed system, the first step can be classified as a Steglich esterification reaction, allowing the production of the intermediate ester at relatively low temperatures [24]. The verification of the successful intermediate production was achieved through H-NMR and FTIR analyses.

**Figure 1:**
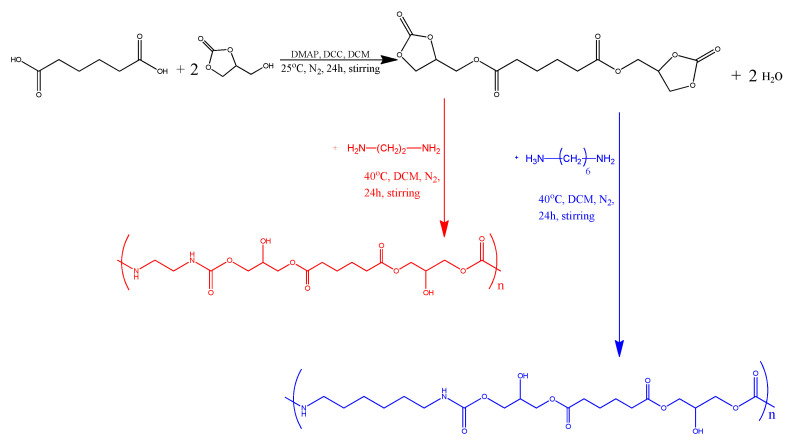
First-step reaction leading to the formation of the intermediate product (ester), and second-step reaction leading to the formation of NIPU_ethyl and NIPU_hexa.

After the first step (esterification) (Figure 1), water was an expected, yet unwanted, byproduct. Thus, moisture was removed from the intermediate (ester) by adding a tablespoon of Na_2_SO_4_ anhydrous to each flask and then removing the precipitate using a vacuum pump and a Buchner funnel. The funnel and the flasks were washed with DCM, and the solvent was collected and supplemented to a final volume of 60 mL. The obtained purified intermediate was then used for a further reaction. In detail, the intermediate was added to a round flask with 1.5 eq. (0.025 mol) of 1,2-ethylenediamine (NIPU_ethyl) (Figure 1) and 1.5 eq. (0.025 mol) of 1,6-hexamethylenediamine (NIPU_hexa) (Figure 1). The reaction took place at 40 °C for 24 h, under stirring and in a N_2_ atmosphere. Two-phase extraction was applied for the purification of the products. In detail, the obtained products were dissolved in DCM (organic phase) and extracted with 1 M HCl (aqueous phase) at a ratio of 2:1. This procedure was repeated twice. The resulting organic phase was left inside the fume hood for three days and was then transferred to an oven under vacuum for 24 h in order to achieve the complete evaporation of the solvent.

### 2.3. Characterization of NIPUs

#### 2.3.1. NMR

The NMR spectra of all the prepared NIPUs were recorded on an Agilent spectrometer (Agilent AM 600, Agilent Technologies, Santa Clara, CA, USA), operating at a frequency of 600 MHz for protons. Deuterated DMSO was used as the solvent. The spectra were calibrated using the residual solvent peak and were analyzed using MestReNova 14.2.0.

#### 2.3.2. Fourier-Transform Infrared Spectroscopy (FTIR)

The chemical structures of the synthesized materials were determined using FTIR (model FTIR-2000, Perkin Elmer, Waltham, MA, USA). The FTIR spectra of all the samples were recorded with an FTIR spectrometer, using KBr disks, in the range 4000–400 cm^−1^ and at a resolution of 2 cm^−1^ (a total of 16 co-added scans). All the spectra were normalized and baseline corrected.

#### 2.3.3. Gel Permeation Chromatography–Size Exclusion Chromatography (GPC/SEC) Analysis

The molecular weights of the materials were determined using gel permeation chromatography–size exclusion chromatography (GPC/SEC) analysis. An Agilent 1260 Infinity II LC system (Agilent Technologies, Santa Clara, CA, USA), equipped with an isocratic G7110B pump, an automatic vial sampler (G7129A), a refractive index detector (RID, G7162A), and a 5 µm PLgel (50 × 7.5 mm) guard column combined with two 5 µm PLgel (300 × 7.5 mm) MIXED-C columns, was employed for the investigation of the molecular weights. The methodology of the standards for the GPC/SEC calibration curves is similar to that in our previous work [25]. The prepared solutions had a concentration of 3 mg/mL and were filtered through 0.45 µm pore size PTFE filters (LabSolutions, Thessaloniki, Greece). The injection volume was 20 μL, and the total elution time of each sample was 30 min. The temperatures of the columns and the RID were both set at 40 °C.

#### 2.3.4. Differential Scanning Calorimetry (DSC)

Conventional differential scanning calorimetry (DSC) was employed for the study of the thermal transitions. The measurements were conducted by means of a TA Q200 apparatus (TA Instruments, New Castle, DE, USA), properly calibrated, on samples of ~11 mg in mass, closed in standard TA aluminum pans in a nitrogen atmosphere and within the temperature range from –100 to 150 °C. Upon erasing the samples’ thermal history by heating at 120 °C at 10 °C/min (scan 1), the samples were cooled down to –100 °C and subsequently heated up to 150 °C at 10 °C/min (scan 2).

#### 2.3.5. Broadband Dielectric Spectroscopy

Broadband dielectric spectroscopy, BDS, was employed for the study of the molecular dynamics, with focuses on the segmental mobility and ionic conductivity effects. A Novocontrol (Novocontrol GmbH, Montabaur, Germany) BDS setup was employed for the investigation, whereas the measurements were carried out in N_2_ (g) flowing on the samples in the form of sandwich-like capacitors of 14 mm in diameter and ~100 μm in thickness (silica spacers employed). The capacitors were prepared by melting the samples (up to ~60–70 °C) between the two disk-like electrodes on a hot plate in order to erase the thermal history, maximize the electrical–thermal contacts, and fix the polymer thickness. The complex dielectric permittivity, ε* = ε′ − i·ε″, was recorded isothermally as a function of the frequency, f, in the range from 10^−1^ to 10^6^ Hz and in the temperature range mainly between −140 and 60 °C during heating at steps of 5 and 10 K, depending on the process followed.

#### 2.3.6. Thermogravimetric Analysis (TGA)

Thermogravimetric analysis (TGA) was carried out in a Setsys 16/18 TG-DTA (Setaram Instrumentation, Lyon, France) instrument. Samples (10 ± 0.5 mg) were placed in alumina crucibles. As a reference, an empty alumina pan was used. The samples were heated from ambient temperature to 600 °C in a 50 mL/min flow of N_2_. A nominal heating rate of 20 °C/min was used, and continuous records of the sample temperature, sample weight, and heat flow were taken. All the measurements were performed in triplicate.

#### 2.3.7. Hydrolysis

The hydrolysis of the prepared NIPUs took place in an aqueous PBS buffer solution (pH 7.4). For enzymatic hydrolysis, *Rhizopus arrhizus* and *Pseudomonas cepacia* lipases were also added to the PBS solution. For this purpose, the mean value of the hydrolysis of the prepared NIPUs was determined by measuring the mass losses of specimens of similar sizes (approximately 1 * 1 cm) and weights in 10 mL of the PBS buffer and PBS–enzyme buffer placed in closed jars and kept at 37 °C. The specimens were taken out every 5 days, washed with water, and dried under vacuum at 60 °C until a constant mass was reached. Each measurement was repeated three times, and as a result, the degree of the hydrolysis of the NIPUs was estimated by the average sample weight loss.

#### 2.3.8. Swelling Capacity

The swelling capacity of the samples was studied in an aqueous phosphate-buffered saline (PBS) solution at pH 7.4. Each sample (1% *w*/*v* and approximately 1 cm × 1 cm) was placed in the buffer solution, and its weight was measured at predetermined time intervals (5, 10, 15, 20, 30, 40, 50, 60, 90, 120, 240, and 360 min). The weighing was carried out after removing the extra moisture brought by the sample. For this purpose, a centrifuge was employed, and the excess water was removed at 4000 rpm for 5 min. Then, using the equations

(1) $$\%\mathrm{swelling}\;\mathrm{ratio}=\frac{w2-w1}{w2}\;*100\%\;$$

where w_1_ = the sample weight at t = 0, and w_2_ = the sample weight at t ≠ 0, and

(2) $$\%\mathrm{water}\;\mathrm{content}=\frac{W\mathrm{wet}-W\mathrm{dry}}{W\mathrm{wet}}\;*100\%\;$$

where w_wet_ = the sample weight after swelling, and w_dry_ = the dry sample weight (before swelling), the swelling ratio and the water content in the prepared samples were calculated.

#### 2.3.9. Solubility at Various pH Levels

For solubility measurements, a pre-weighed amount of each material, with approximately 1 cm × 1 cm dimensions, was added to an aqueous PBS buffer solution at pH levels of 3, 4, 6, 7, 10, and 11 to a final polymer content of 1% *w*/*v*. The solutions were left at 30 °C for 24 h inside a mechanical shaker. The next day, the samples were placed in a vacuum oven (60 °C, 200 mbar) for another 24 h, and then the samples were weighed. The solubility percentage (%weight loss) was calculated from the weight of the solid sample.

## 3. Results and Discussion

### 3.1. NMR

**Figure 2:**
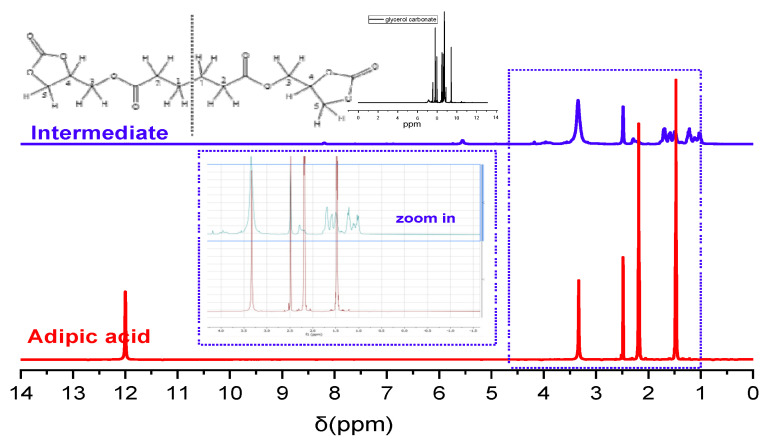
H-NMR spectra of adipic acid and the intermediate.

The intermediate product and both NIPUs were characterized using ^1^H-NMR spectroscopy to confirm their successful synthesis and verify their structures. In all the spectra, the peak at approximately 2.5 ppm corresponds to DMSO. The spectrum of the intermediate includes peaks at 1.5–2 ppm, which correspond to H in CH_2_ groups (H1 and H2 in the structural depiction in Figure 2). The peaks at 3.25 and 3.9 ppm correspond to H in the groups CH_2_-O and CH-O (H3 and H5 and H4, respectively). The confirmation of the first step’s success derives mainly from the fact that no peaks are detected at 12 ppm, indicating that there are no OH groups in the intermediate product. This absence suggests that the carboxylic groups of adipic acid reacted efficiently with the hydroxyl groups of glycerol carbonate molecules. The peaks that belong to the CH_2_-O and CH-O hydrogens further support the successful intermediate synthesis (Figure 2 “zoom-in” view).

**Figure 3:**
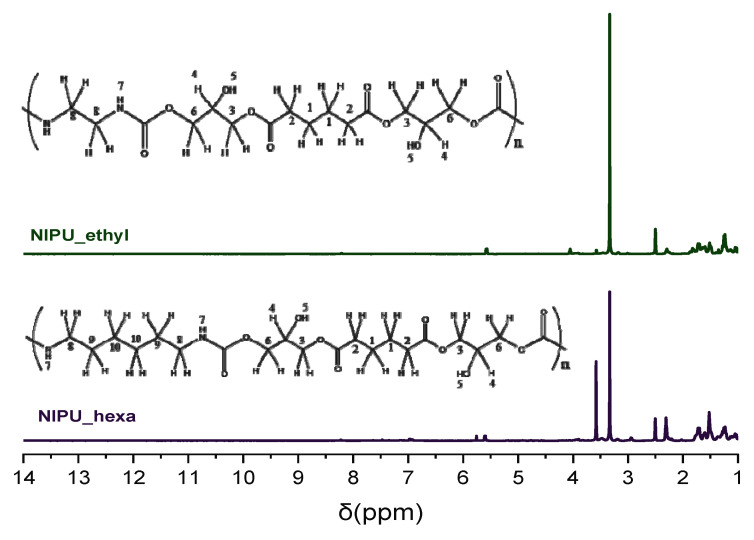
H-NMR spectra of NIPU_ethyl and NIPU_hexa.

The NMR spectral analysis of NIPU_ethyl shows peaks from 1.25 to 3 ppm, which correspond to protons in methylene and methine groups (CH_2_ and CH). At 1.25 ppm, the peak corresponds to H1; at 1.7 ppm, it corresponds to H8; at 2.25 ppm, it corresponds to H2; and at 3 ppm, it corresponds to H4. The peaks at 3.4 and 4 ppm belong to H in methylene groups, which have a single bond with oxygen, CH_2_-O (H3 and H6, respectively). At approximately 5.6 ppm, one can observe a peak that is attributed to H in the hydroxyl group (OH) (H5), and at 7–8.2 ppm, the peak that appears belongs to the hydrogens in the amine group, which is next to the carbonyl NHC=O group (H7) (structural description in Figure 3 (top)).

Similarly, in the H-NMR spectrum of NIPU_hexa, the peak at 1.25 ppm corresponds to the methylene groups’ hydrogens (H1), the peak at 1.7 ppm belongs to the methylene groups’ hydrogens (H10), and the peaks at 2.3, 2.9, and 3.25 ppm correspond to the methylene groups’ hydrogens (H9, H2, and H8). The H in the methine groups (CH) (H4) is detected at 3.4 ppm. The peaks at 3.5 and 3.9 ppm are attributed to the Hs in methylene groups that are next to oxygen via a single bond (CH_2_-O) (H3 and H6). Furthermore, the peak at 5.5 ppm belongs to the H in the hydroxyl groups (OH) (H5), and the one at 7–8 ppm corresponds to the H in the amino groups that are next to a carbonyl group (NHC=O) (H7) (structural description in Figure 3 (bottom)). The aforementioned data, especially the peaks corresponding to the H in the NHC=O groups, verify the successful synthesis of the NIPUs.

### 3.2. Fourier-Transform Infrared Spectroscopy (FTIR)

**Figure 4:**
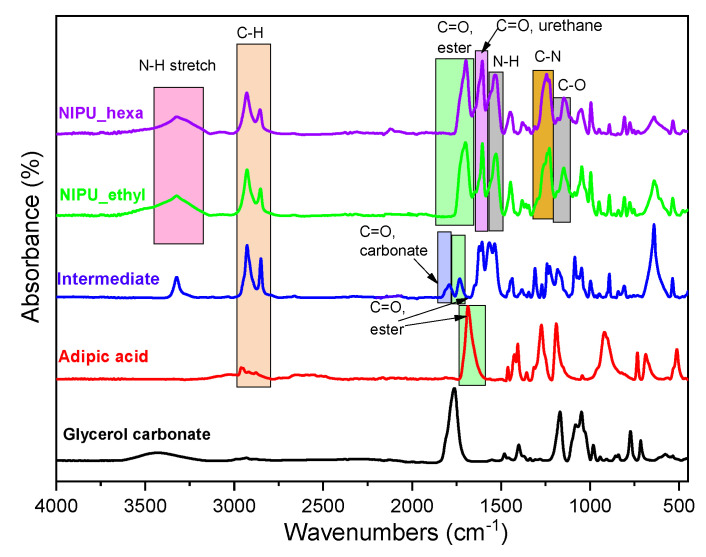
FTIR spectra of adipic acid, the intermediate product, NIPU_ethyl, and NIPU_hexa.

The FTIR spectra of the prepared materials are presented in Figure 4. First, the spectrum of the intermediate product shows characteristic peaks at 1730 cm^−1^ (carbonyl C=O in ester groups belonging to the main chain of the adipic acid), 1790 cm^−1^ (carbonyl C=O in the carbonate group, -O-C(O)-O-, of the glycerol carbonate molecule), 2845 cm^−1^, and 2930 cm^−1^ (C-H bond in alkyl groups). The absence of peaks belonging to hydroxyl groups is a strong indication of the formation of ester bonds between the hydroxyl groups of glycerol carbonate molecules and the carboxylic groups of adipic acid. These results further complement the obtained NMR analysis results, indicating the success of the first step’s synthesis.

Concerning the final products, in the spectrum of the NIPU_ethyl, peaks were observed at 1145 cm^−1^ (C-O single bond), 1230 cm^−1^ (C-N single bond), 1530 cm^−1^ (N-H bonds’ bending vibrations), 1605 cm^−1^ (carbonyl C=O in the urethane group), 1700 cm^−1^ (carbonyl C=O in ester groups belonging to the initial carboxylic ones of adipic acid), 2930 cm^−1^ (C-H bonds in alkyl groups), and 3325 cm^−1^ (N-H bonds’ stretching vibrations), and a broad peak was observed from 3145 to 3635 cm^−1^ (O-H bonds of hydroxyls that appeared because of the opening of the glycerol carbonate). Additionally, NIPU_hexa presents very similar FTIR peaks compared to those of NIPU_ethyl. In this case, NIPU’s production is verified mainly by the presence of the peaks belonging to the N-H bond (stretching and bending vibrations), the C-N single bond, and the two different C=O bonds (of the urethane and of the ester groups).

### 3.3. Gel Permeation Chromatography (GPC)

To confirm the molecular weights of NIPUs, GPC analysis was carried out, as shown in Table 1. The molecular weight of PU is influenced by several factors (the monomers, molar ratio of the diisocyanate to the polyol, and reaction conditions). Similar molecular weight characteristics of polymers have also been reported by numerous other research groups [26,27,28].

### 3.4. Differential Scanning Calorimetry (DSC)

**Figure 5:**
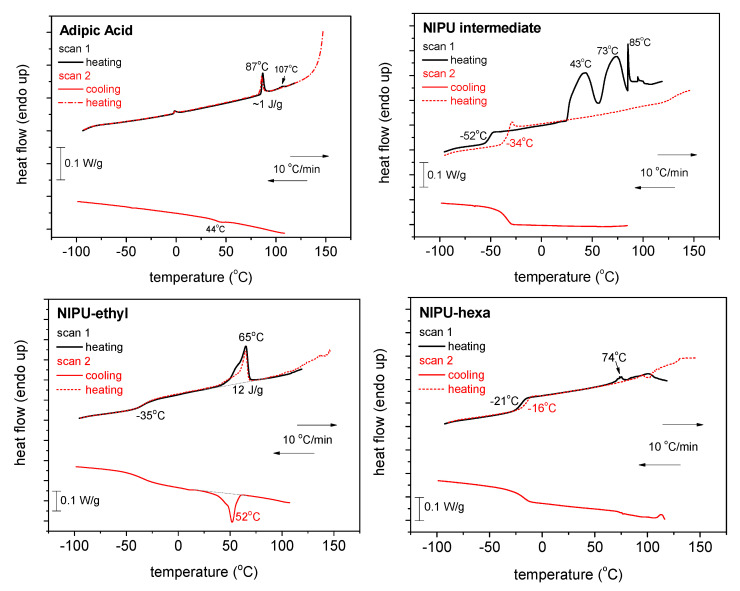
Comparative DSC traces for all the samples and scans performed. The heat flow is presented upon normalization to each sample’s mass.

**Figure 6:**
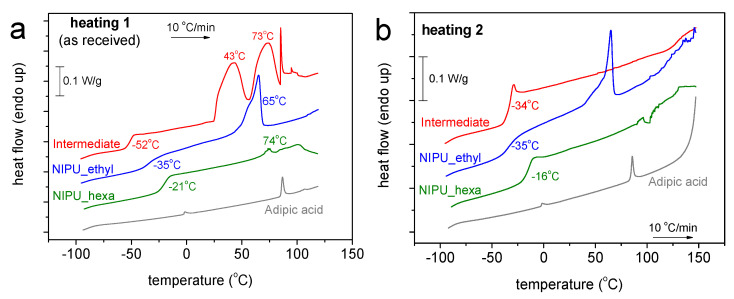
Comparative DSC traces for all the samples during heating in (**a**) scan 1 and (**b**) scan 2. The heat flow has been normalized to the sample’s mass.

The results obtained using DSC can be seen in Figure 5 and Figure 6, for all the samples and for both scans 1 and 2. In Figure 5, we show comparative plots for the four samples of the heating scans for scan 1 and scan 2. The plot of the initial adipic acid exhibits mainly two weak endothermal peaks during heating at 87 and 107 °C, possibly arising from the melting of crystals. The corresponding crystallization (a weak peak as well) is recorded during cooling at around 44 °C. The trace of the intermediate product exhibits a glass transition step, initially at −52 °C (scan 1) and migrates to −34 °C upon the second heating. At higher temperatures, during scan 1, a variety of strong endothermal peaks are recorded, including a sharp peak at 85 °C, resembling the peak corresponding to the melting point of the adipic acid. Interestingly, the endothermic peaks vanish in scan 2, suggesting that the intermediate may include some impurities or the solvent. The recorded alternation in T_g_ could be because of the remaining solvents in addition to the less thermally stable characteristic expected for the said sample. During cooling, the DSC trace of the intermediate does not exhibit any crystallization peak.

Regarding NIPU_ethyl, this material demonstrates the behavior of a semicrystalline polymer; namely, the trace shows a crystallization peak at ~52 °C, a glass transition peak at −35 °C, and a relatively weak peak corresponding to melting at ~65 °C. The thermal events do not change between the two scans. A similar behavior is recorded for NIPU_hexa, which exhibits, however, peaks at elevated T_g_ values (−21 and −16 °C), a peak at an elevated melting temperature (74 °C), and quite weaker crystallizability.

One of the main calorimetric findings is the improved thermal stability observed in NIPU_ethyl and NIPU_hexa, when compared with that of the NIPU intermediate. This thermal stability improvement should be connected with the more rigid characteristic of the said samples (elevated T_g_ values) [29].

### 3.5. Broadband Dielectric Spectroscopy Results

**Figure 7:**
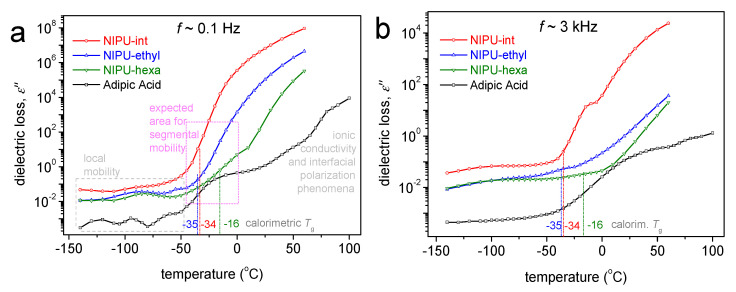
Comparative isochronal ε”(T) plots for all the samples, shown at (**a**) a relatively low frequency of 100 mHz, namely, closer to the equivalent frequency of the DSC at T_g_, and (**b**) a higher frequency of ~3 kHz to suppress the extensive contribution of the conductivity at the higher T. Marked in both figures are the values of the calorimetric T_g_ for heating in scan 2.

To provide a more direct comparison of the dielectric signal with that obtained using calorimetry, we present in Figure 7, the temperature dependence of the imaginary part of the dielectric permittivity (ε”) at two selected frequencies. Since ε” is considered to represent the dielectric loss well, any dipolar relaxation mechanism arising from actual molecular motions is recorded as the peak of ε”, at least for temperatures below and slightly above T_g_ [30]. The main relaxation of interest is the one called α relaxation, the dielectric analog of the glass transition. For the samples under investigation, the dielectric recordings do not allow the firm detection of the α relaxation. However, there are indirect indications for its effects on the glass transition temperature. For example, at a relatively low frequency of 0.1 Hz (Figure 7a), which is not far from the equivalent frequency of the DSC, the dielectric signal rises or tends to strongly increase at T values close to the calorimetric T_g_.

The effects related to ionic conductivity, i.e., the transport of free charges (ions) throughout the polymer matrix, occur at higher temperatures. Obviously, the charges may mobilize within a soft (liquid-like) phase, and this occurs in a polymer at T > T_g_. In the NIPUs, the initial T for ionic conductivity development increases in the order of NIPU-intermediate < NIPU-ethyl < NIPU-hexa. The latter order does not actually coincide with that of the increasing T_g_ (NIPU-ethyl ≤ NIPU-int < NIPU-hexa). Because it is known that PUs often exhibit a phase separation, e.g., between their soft and hard domains [31], we expect that this discrepancy could be not simply because of the T_g_ alternation (of the soft domains) but also because of the degree of the phase separation as well as the concentrations of free ions within the soft domains. Such ions could artificially exist in polymers, from the remaining solutions or other impurities. This is more or less expected in the NIPU-intermediate and is compatible with the corresponding DSC results (scan 1), as mentioned in the previous section.

### 3.6. Thermogravimetric Analysis (TGA)

**Figure 8:**
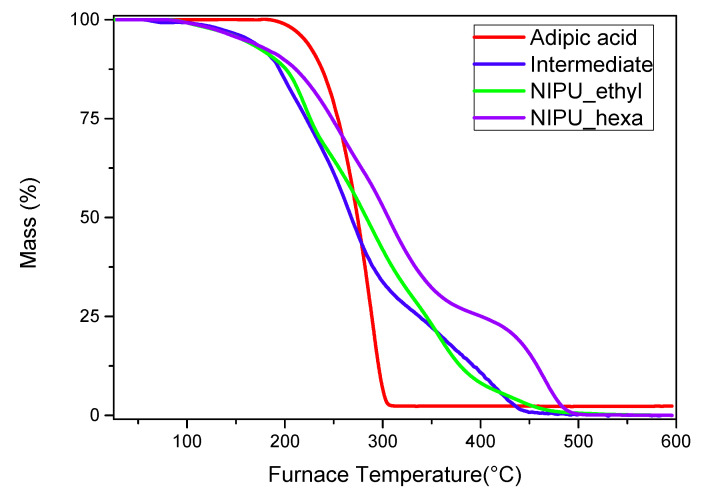
TGA thermogram (mass loss (%)–temperature (°C)) graphs for adipic acid, the intermediate, NIPU_ethyl, and NIPU_hexa.

The TGA analysis results showed that the intermediate product, NIPU_ethyl, and NIPU_hexa start decomposing at lower temperatures compared to that of neat adipic acid (Figure 8). In detail, the intermediate’s decomposition starts at ~130 °C and shows a second step at ~400 °C. At the maximum temperature (~450 °C), the remaining percentage of the mass is zero (0%). Both NIPU samples also exhibit weight losses around 100–200 °C; however, they are attributed mainly to the vaporization of the residual and linked molecules of water. NIPU_ethyl’s actual decomposition starts at ~250 °C and takes place in one step. The remaining mass percentage is zero at the highest temperature (450–500 °C). NIPU_hexa’s decomposition takes place in one step close to 300 °C. The maximum temperature, at which the zero mass-percentage is recorded, is 500 °C. These results are in accordance with the literature, as many scientific papers have proved that the final decomposition step and the end of the overall degradation of NIPUs happen in the range 425–525 °C [32,33,34,35,36].

**Figure 9:**
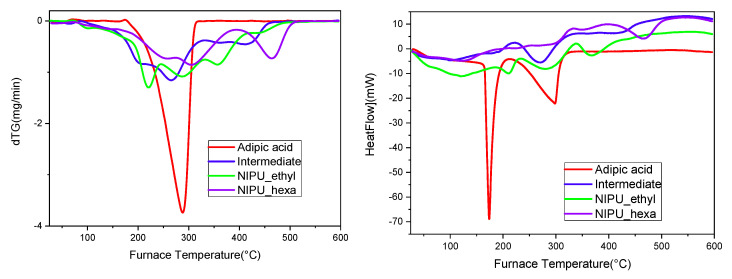
(**Left**) DTG–temperature (°C) and (**right**) heat flow–temperature graphs (thermograms) for adipic acid, the intermediate, NIPU_ethyl, and NIPU_hexa.

Furthermore, the multiple peaks that appear in the DTG–temperature graph (Figure 9, left) have been previously reported not only by Carré, Bonnet, and Avérous but also in the work of Chen, Pizzi, and Fredon et al. Usually, the first peak/curve (around 100–200 °C) corresponds to the vaporization of the residual and linked water molecules. The degradation of the urethane linkages, including the ester bonds, is spotted approximately in the range 200–400 °C and is often described as a two-step process, with the first degradation stage at 250–280 °C (the degradation of urethane’s hard segment, depending on the specific structure of the material and the surrounding environment) and the second at 350–450 °C (the degradation of the ester group (soft segment), depending on the specific structure of the material and the surrounding environment). However, these two steps tend to overlap [37]. The curves around the values of 400–500 °C are attributed to the decomposition of the carbonated chains (the breakage of the C-C covalent bonds), and it is not uncommon for them to overlap with the urethane decomposition stage [32,36]. According to the literature, the degradations of various NIPUs have been reported as two-step, one- step, or even multistage processes, therefore leading to the conclusion that the thermal decompositions of these materials are strongly dependent on the types of cyclic carbonate and diamine employed during synthesis [37].

### 3.7. Hydrolysis–Enzymatic Hydrolysis

**Figure 10:**
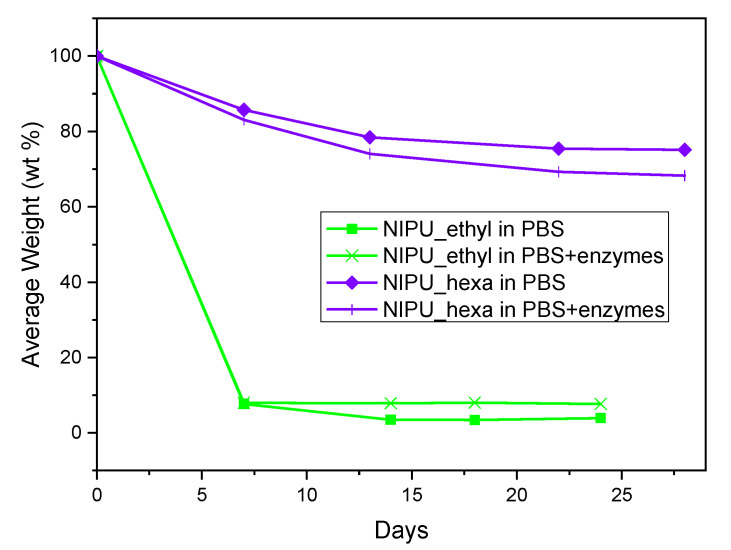
Hydrolysis and enzymatic hydrolysis of NIPU_ethyl and NIPU_hexa.

The results of the hydrolysis and enzymatic hydrolysis of NIPU_ethyl and NIPU_hexa are presented in Figure 10. NIPU_ethyl lost the majority of its mass (93–97%) from day 0 to day 7 and reached approximately 3% (PBS) and 7% (PBS with enzymes) average mass losses on day 18. At the beginning of the measurements, the NIPU_ethyl samples that underwent hydrolysis exhibited a very similar behavior to that of the samples that underwent enzymatic hydrolysis. However, during the last 14 days, an increased average weight loss percentage was observed in NIPU_ethyl’s enzymatic hydrolysis compared to its hydrolysis without enzymes. In contrast, NIPU_hexa had, evidently, a more rapid hydrolysis in the presence of enzymes. Additionally, its mass loss was not as significant as NIPU_ethyl’s, considering the fact that during the last day of the measurement, NIPU_hexa retained approximately 75% (PBS) and 68% (PBS with enzymes) of its initial mass. Consequently, it was concluded that NIPU_hexa is notably more stable in aqueous environments than NIPU_ethyl. A plausible explanation for this could lie in the fact that according to the literature, hydrolytic resistance increases with an increase in the material’s hydrophobicity [38]. On that note, it has been reported that the various amines employed in NIPU synthesis could determine the hydrophilic/hydrophobic balance of the obtained material [28]. In fact, NIPUs obtained using the same dicarbonate but different diamine molecules were studied, and the results showed an increased contact angle with water when 1,6-hexamethylenediamine was utilized in comparison to that when 1,2-ethylenediamine was utilized [39]. Accordingly, NIPU_hexa, which includes a longer carbon (hydrophobic) chain than NIPU_ethyl because of the employment of 1,6-hexamethylenediamine, is expected to present higher hydrophobicity and, therefore, higher resistance to hydrolysis than NIPU_ethyl. Molecular weight differences between NIPU_ethyl (5380) and NIPU_hexa (7290) could also contribute to the observed differences in the hydrolysis behavior, as it is common for higher-molecular-weight polymers to exhibit reduced hydrolysis rates because of their lower solubilities and decreased chain mobilities, which may hinder water penetration and enzymatic attack.

### 3.8. Swelling

**Figure 11:**
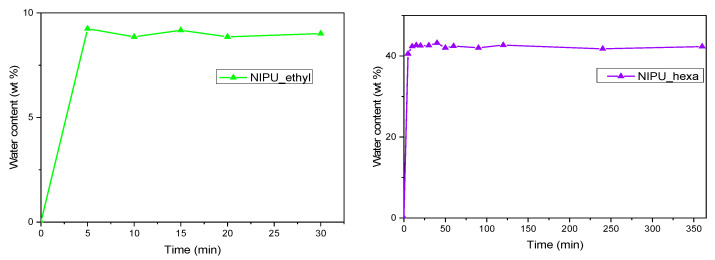
Swelling capacity in terms of the water contents of NIPU_ethyl and NIPU_hexa.

In order to determine the swelling behaviors of the NIPUs, graphs depicting the percentage of the water content in relation to time were plotted (Figure 11). The graph for NIPU_ethyl revealed that the material absorbs water during the first 30 min. Further on, not only the water uptake stops but also the polymer starts losing mass (Figure 11, left). To further highlight this effect, a graph depicting NIPU_ethyl’s weight in relation to time has been added to the swelling diagram. As we observe, between 30 and 60 min, NIPU_ethyl violently loses weight, and from then on, the mass of the material remains practically constant.

In contrast, the swelling graph for NIPU_hexa shows two stages: the first stage (0–40 min) is connected to the rapid absorbance of water, and the second stage (40–300 min) corresponds to an equilibrium, where the material stops absorbing water, and the water percentage practically remains constant. Similar behavior has been recorded in NIPU swelling tests [40]. In more detail, once the maximum value (43%) was reached, it stayed stable for the next 300 min (the final measurement was at 360 min). This behavior has been recorded in other scientific articles, too, with the maximum value of the water uptake and the time varying for each different NIPU [40,41].

The differences in the swelling behaviors that NIPU_ethyl and NIPU_hexa exhibit could be explained by a statement that Catalá, Guerra et al. expressed in their work [6]. They commented that shorter amines (meaning those with fewer CH_2_ units in their chains) lead to NIPUs with higher concentrations of intramolecular hydrogen bonding because of the shorter distances between the groups that participate in these bonds, something that reduces the polymer’s ability to swell. In addition, Naheed, Zuber et al. mentioned a similar theory in their scientific article about polyurethane elastomers that contain macrodiols of varying molecular weights [41].

### 3.9. Solubility at Various pH Levels

**Figure 12:**
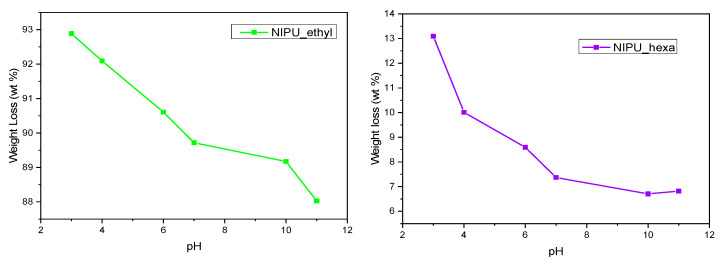
Weights of NIPU_ethyl and NIPU_hexa at various pH levels.

The solubilities of the NIPUs in different-pH solutions are presented by comparing the weight loss percentages in relation to the pH values of the NIPUs’ liquid environments. Both NIPUs exhibit the same behavioral pattern: the weight loss percentage increases in acidic solutions and decreases as the pH becomes more basic (Figure 12). Similar results have been reported by Gennen, Sandro et al. in their work on pH-sensitive NIPU copolymers [42]. However, the weight loss percentage for NIPU_ethyl (88–93%) is much higher than that for NIPU_hexa (6.5–13%). A potential explanation for this phenomenon lies in the fact that NIPU_ethyl appears to be a lot more hydrophilic and susceptible to degradation in aqueous environments than NIPU_hexa. As a result, the presence of hydrogen ions at acidic pH levels enhanced the hydrolysis, resulting in a faster and more effective hydrolysis for NIPU_ethyl compared to that for NIPU_hexa.

## 4. Conclusions

In the present work, the successful production of NIPUs was achieved through the polyaddition path, utilizing adipic acid, glycerol carbonate, 1,2-ethylenediamine, and 1,6-hexamethylenediamine, resulting in NIPU_ethyl and NIPU_hexa. The obtained products were investigated in detail in terms of structures and properties. Spectroscopic analyses, including H-NMR and FTIR analyses, verified the production of the intermediate and the NIPUs. In more detail, H-NMR confirmed the synthesis of the intermediate by the absence of peaks at 12 ppm (OH groups) and by the presence of H peaks corresponding to NHC=O groups. FTIR spectroscopy further verified the structures of the NIPUs through the detection of peaks corresponding to C=O of the urethane and ester groups, the peak attributed to the bending vibrations of the N-H bond, and the peak corresponding to the C-N bond. TGA analysis revealed that the thermal degradation of the intermediate product and NIPU_hexa takes place in two steps, while NIPU_ethyl degrades in one step. All the materials have a final zero mass-percentage at their maximum temperatures (450–500 °C). The facilitated thermal stabilities of NIPU_ethyl and NIPU_hexa, as compared to that of the intermediate product, which was one of the scopes herein, are additionally confirmed by the calorimetric and dielectric results (molecular mobility and ionic conductivity at high T values). Both techniques provide proof that the two said NIPUs exhibit more rigid structures in addition to being better purified, the latter actually being achieved via the corresponding synthetic route. Swelling and solubility tests at various pH levels were also performed. The swelling tests revealed that NIPU_hexa presents enhanced absorption capacity compared to NIPU_ethyl. Furthermore, both materials showed increased mass losses in acidic environments during the solubility tests at different pH levels; however, their mass loss percentages were significantly different. A possible explanation could be that at acidic pH levels, NIPU_ethyl is more hydrophilic than NIPU_hexa, which resulted in a more effective hydrolysis rate. Lastly, the hydrolysis and enzymatic hydrolysis of NIPUs reinforced the observation that NIPU_ethyl tends to hydrolyze rapidly because its mass appears to be 3–7% on day 18, in comparison to NIPU_hexa, which retained almost 70% of its mass during the last measurement. The differences between the molecular structures of NIPU_ethyl and NIPU_hexa arise primarily from the different diamines used in their synthesis—1,2-ethylenediamine for NIPU_ethyl and 1,6-hexamethylenediamine for NIPU_hexa. The shorter, more polar ethylene chain in NIPU_ethyl leads to increased hydrophilicity, which is reflected in its rapid hydrolysis and lower thermal stability than that of NIPU_hexa. In contrast, NIPU_hexa, with its longer aliphatic hexamethylene chain, exhibits higher hydrophobicity, greater thermal stability, and slower hydrolysis than those of NIPU_ethyl, as supported by swelling, solubility, and enzymatic hydrolysis tests.

Taking this research a step further, NIPUs could be tested in terms of cytotoxicity, while, additionally, antimicrobial tests could be performed in order to investigate their potential use as drug loading/release system complexes.

## Figures and Tables

**Table 1:** GPC analysis of the synthesized NIPUs.

Name	Diamine	Molecular Weight (Mw)	PDI
NIPU_ethyl	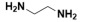	5380	1.00
NIPU_hexa	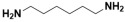	7290	1.00

## Data Availability

The original contributions presented in this study are included in the article. Further inquiries can be directed to the corresponding author.
